# Independent value of serum *β*-human chorionic gonadotropin in predicting early pregnancy loss risks in IVF/ICSI cycles

**DOI:** 10.3389/fimmu.2022.992121

**Published:** 2022-09-29

**Authors:** Liyan Wang, Yanbiao Jiang, Haofei Shen, Xiaoling Ma, Mingxia Gao, Panpan Jin, Rui Zhang, Lihui Zhao, Xuehong Zhang

**Affiliations:** ^1^ Reproductive Medicine Center, The First Hospital of Lanzhou University, Lanzhou, China; ^2^ Key Laboratory for Reproductive Medicine and Embryo, Lanzhou, China; ^3^ The First School of Clinical Medicine, Lanzhou University, Lanzhou, China

**Keywords:** human chorionic gonadotropin, early pregnancy loss, IVF/ICSI cycle, prediction, risk factors

## Abstract

**Background:**

Early pregnancy loss (EPL) is the most prevalent complication, particularly in couples undergoing assisted reproductive technology treatment. The present study aimed to determine whether the serum *β*-human chorionic gonadotropin (*β*-hCG) level after 14 days of embryo transfer, either alone or in conjunction with other parameters in IVF/ICSI cycles, could be used to predict subsequent EPL.

**Methods:**

This was a retrospective cohort study of all couples who received clinical pregnancy and underwent fresh IVF/ICSI cycles at a single large reproductive medical center between January 2013 and June 2020. The research involved a total of 6600 cycles. For risk variables, we conducted the least absolute shrinkage and selection operator (LASSO) analysis, and for risk scoring, we used logistic regression coefficients. To analyze relevant risk factors for EPL, univariate and multivariate logistic regression analyses were employed. Areas under the curve (AUC) were determined and compared between β-hCG and other factors using receiver operating characteristic (ROC) curves.

**Results:**

*β*-hCG level was considerably lower in women who had EPL than in those who were ongoing pregnancy (564.03 ± 838.16 vs 1139.04 ± 1048.72 IU/L, p< 0.001). Univariable and multivariable logistic regression revealed that *β*-hCG levels were significantly correlated with the probability of EPL, independent of other risk factors. More importantly, the *β*-hCG level could independently predict the occurrence of EPL and was comparable to the model that combined other risk factors. Th*e* optimal serum *β*-hCG cut-off value for predicting EPL was 542.45 IU/L.

**Conclusions:**

Our results suggest that the serum *β*-hCG level has a strong independent predictive value for EPL occurrence in fresh IVF/ICSI cycles.

## Introduction

Pregnancy loss is common, affecting 15.3% of clinically recognized pregnancies (1). Most pregnancy losses (85 %) occur before the 12th week of pregnancy, known as early pregnancy loss (EPL). Furthermore, patients receiving assisted reproductive technology (ART) have a high EPL ratio of 14.7% (2). Numerous studies have focused over the years on factors related to EPL, such as embryonic chromosomal (3), diabetes (4), endocrine, reproductive immune (5), infection (6), and maternal-fetal interface (7); however, in several cases, no cause could be found. Moreover, the occurrence of EPL will result in poor results, including significant physical and psychological stress and significant economic burden, specifically in the setting of desired pregnancy achieved *via* ART. Hence, both patients and physicians are eager for a method for EPL prediction and prevention. The factors that predict EPL are not fully understood at this time.

Human chorionic gonadotropin (hCG) is a glycoprotein hormone that reproduction. HCG is composed of two glycosylated subunits, α-(93-amino acid, 14.5 kDa) and β-(145-amino acid, 22.2 kD). The α-subunit is homologous to pituitary luteinizing hormone(LH), follicular stimulating hormone (FSH), and thyroid-stimulating hormone(TSH). The β-subunit of hCG is specific and responsible for hCG’s biological activity (8). Additionally, the 24 amino acids at the N-terminus of the β-subunit are unique to hCG (8). hCG is mainly secreted by trophoblasts in early pregnancy and exerts various effects in the establishment and maintenance of pregnancy, including maternal immune tolerance at the maternal-fetal interface (9), trophoblast invasion (10), decidualization (11), and promotion of angiogenesis (12). Furthermore, hCG modulates endometrial receptivity by regulating multiple related cytokines (13). HCG is implicated in influencing various immune cells known to play an essential role in embryo implantation and pregnancy maintenance, such as natural killer (pNK) cells (14), regulatory T-cell cells (Tregs) (15), and dendritic cells(DCs). Currently, human chorionic gonadotropin (hCG) is broadly used for early pregnancy diagnosis and monitoring.

Although there is an association between serum hCG levels and pregnancy, there is no ideal cut-off value for EPL prediction. Additionally, many researchers have focused on various risk factors for EPL, such as parental age (1), body mass index (BMI) (16), ultrasound measurements (17), and other clinical factors. Therefore, we investigated whether β-hCG combined with other factors could improve EPL’s predictive value.

The purpose of this study was to explore the risk factors of EPL in patients who underwent clinical pregnancy following fresh cycles *in vitro* fertilization (IVF) or intracytoplasmic sperm injection (ICSI) treatment, as well as to clarify the ability of serum β-hCG level 14 days after transfer to predict EPL based on retrospective data analysis. These results can help medical staff to take measures to minimize pregnancy loss after IVF/ICSI treatment, as well as aid in decision-making.

## Materials and methods

### Study population

**Figure 1:**
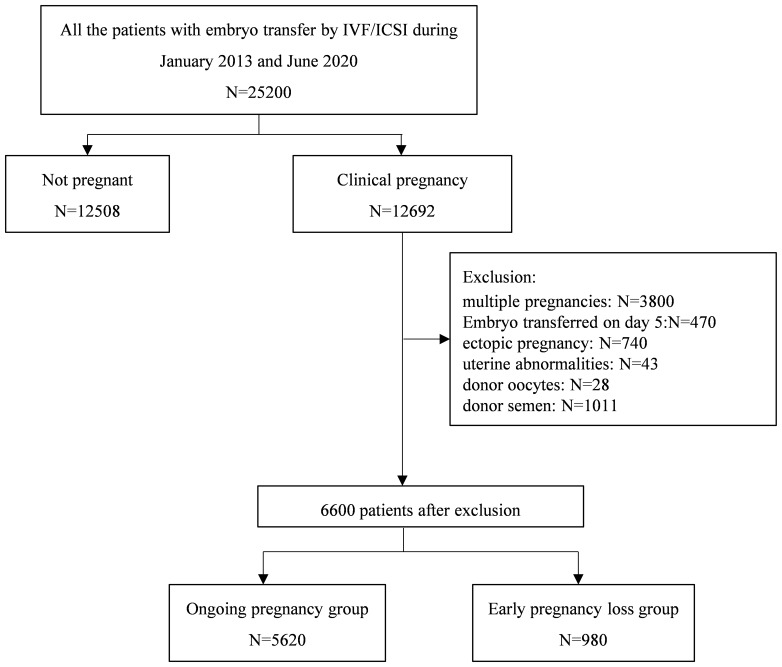
Flow chart of the research.

In this retrospective study, the enrolled patients had received clinical pregnancies and were undertaking IVF/ICSI cycles at the reproductive center of the First Hospital of Lanzhou University. The medical records of all patients who conceived from January 2013 to June 2020 by IVF/ICSI treatment were screened. Only those patients with cleavage-stage embryo transfer (on day 3) and those with singleton pregnancies were included in this study. The study exclusion criteria included multiple pregnancies with more than one gestational sac detected on ultrasound, ectopic pregnancy, pre-implantation genetic testing, uterine abnormalities (such as endometrial polyps, submucous uterine fibroids, adenomyoma, uterine malformations, untreated septate uterus, or untreated intrauterine adhesions), as well as those involving donor oocytes or semen. The flow diagram of patient selection is depicted in **Figure 1**. HCG was not used in all luteal phase-support protocols. The indications for IVF/ICSI included tubal, endocrine, immune, and male factors.

### Outcome variables

A total of 6600 clinic pregnancy patients were assigned to 2 groups based on their early pregnancy outcomes, as follows: the ongoing pregnancy group (>12-week gestation) and the EPL group. Clinical pregnancy was defined as the presence of a gestational sac on ultrasonography. The primary outcome was EPL, which was defined as pregnancy loss before the 12th gestational week (after 67 days of embryo transfer). Pregnancy was defined as the presence of a gestational sac and detection of a fetal heartbeat after 12 weeks of gestation.

Clinical and laboratory data were extracted directly from the electronic medical records, which included maternal age, paternal age, maternal BMI, the duration of infertility, infertility type, total antral follicle count (AFC), the number of retrieved oocytes, the number of metaphase-2 oocytes, the fertilization methods, endometrial thickness, the number of transferred embryos, estradiol/progesterone ratio (E2/P) on the day of ovulation trigger, and the serum β-hCG levels. The β-hCG levels were measured 14 days after transfer at a single laboratory.

### Statistical analysis

Data analysis was performed using the EmpowerStats statistical software (X&Y Solutions). Continuous variables were expressed as the mean ± standard deviation (SD) and categorical variables as N (%). Univariable and multivariable logistic regression analyses were performed to evaluate the relevant risk factors for EPL. The R package “glmnet” was employed to perform Least Absolute Shrinkage and Selection Operator (LASSO) binary logistic regression analyses, while the “rms” package was used to create the nomogram. Receiver operating characteristic (ROC) curves were plotted, and the areas under the curve (AUC) were calculated and compared between β-hCG and other factors. The optimal cut-off values were estimated by using the Youden index.

## Results

**Table 1** depicts the general characteristics of the women with ongoing pregnancies and those with EPL. This research comprised a total of 6600 clinical pregnancy cycles, 980 of which met EPL. Except for fertilization methods, there were significant differences in general characteristics between the two groups, including maternal age, paternal age, number of retrieved oocytes, duration of infertility, maternal BMI, total AFC, number of metaphase -2 oocytes, endometrial thickness, serum β-hCG levels, E2/P, and type of infertility. EPL Patients had higher maternal age, paternal age, infertility duration, maternal BMI, and secondary infertility rate. In contrast, the EPL group had fewer retrieved oocytes, total AFC, metaphase-2 oocytes, endometrial thickness, and E2/P than the ongoing pregnancy group. Serum β-hCG levels were markedly lower in EPL patients than in ongoing pregnancy patients [564.03 ± 838.16 vs. 1139.04 ± 1048.72; P < 0.001].

**Table 1:** The characteristics of cycles in the study population(N=6600).

	Ongoing pregnancy (n = 5620)	Early pregnancy loss (n = 980)	P value
Maternal age (y)	30.39 ± 4.04	31.74 ± 4.81	<.01
Paternal age (y)	32.07 ± 4.80	33.51 ± 5.64	<.01
Maternal BMI (kg/m2)	22.20 ± 3.11	22.46 ± 3.01	<.05
Infertility duration (y)	3.83 ± 2.80	4.17 ± 3.24	<.01
Infertility type			<.01
Primary	3320 (59.33%)	523 (53.64%)	
Secondary	2276 (40.67%)	452 (46.36%)	
AFC	14.99 ± 6.74	14.54 ± 7.33	<.01
Number of retrieved oocytes	13.98 ± 6.26	13.01 ± 6.30	<.01
Number of metaphase-2 oocytes	12.69 ± 6.01	11.88 ± 6.17	<.01
Fertilization methods			.64
IVF	3489 (62.08%)	616 (62.86%)	
ICSI	2131 (37.92%)	364 (37.14%)	
Endometrial thickness (cm)	1.13 ± 0.21	1.11 ± 0.22	<.01
Number of transferred embryos			.19
1	1572 (27.97%)	198 (20.20%)	
2	3529 (62.79%)	733 (74.80%)	
3	519 (9.23%)	49 (5.00%)	
E2/P	3.12 ± 2.16	2.91 ± 2.18	<.01
β-hCG (IU/L)	1139.04 ± 1048.72	564.03 ± 838.16	<.01

Values are mean±standard deviation or number (percentage)

AFC, total antral follicle count; BMI, body mass index.

**Figure 2:**
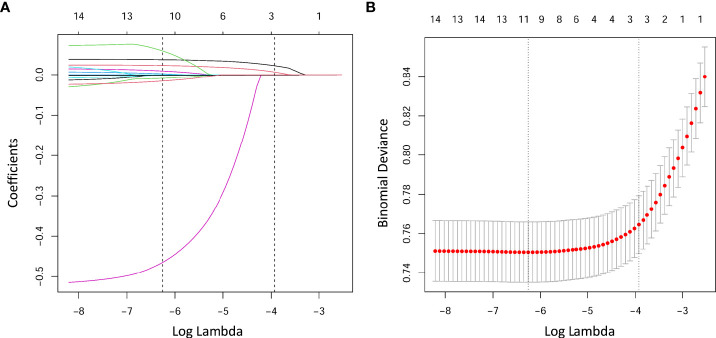
Lasso regression analysis of factors related to EPL. **(A)** LASSO coefficient profiles of the factors. The vertical line was drawn at the value selected by 10-fold cross-validation, where the optimal λ resulted in 13 nonzero coefficients. **(B)** The lambda (λ) selection process in the LASSO regression is depicted. The value of λ that gave the minimum average binomial deviance was applied to select the features. Dotted vertical lines were drawn at the optimal values using the minimum criteria and the 1 − SE criteria. The optimal value of 0.0019 with a log(λ) value of -6.2754 was obtained.

The LASSO logistic regression analysis was conducted to comprehensively analyze the role of all the variables on prediction. We achieved two EPL risk models using LASSO regression analysis. The first is based on lambda. min corresponding to the minimum mean error while the second is on lambda.1se, that is, the maximum lambda corresponds to the minimum mean error within one standard deviation (**Figure 2A**). **Figure 2B** represents the corresponding relationship between coefficients of each risk factor and the lambda. In addition, we defined the radscore and the radscore formula of the radiomics as follows:


$$\begin{array}{c} Radiomics\;score=0.03736\times X1\;+\;0.02298\times X2-0.00858\times X3\;+\;0.00222\times X5\;+\;0.0092\times X7\;+\;0.05919\times X9.0-0.46504\times X11-0.00138\times \end{array}\begin{array}{c} X12-0.01533\times X14 \end{array}$$

In the equation above, X1 represents maternal age, X2 represents paternal age, X3 represents the number of retrieved oocytes, X5 represents infertility duration, X7 represents AFC, X9.0 represents infertility type (IVF), X11 represents endometrial thickness, X12 represents β-hCG, and X14 represents E2/P.

Maternal age, paternal age, number of retrieved oocytes, duration of infertility, maternal BMI, number of metaphase-2 oocytes, endometrial thickness, E2/P, secondary infertility rate, and the β-hCG level were associated with the risk of EPL in a univariable logistic regression analysis (**Table 2**). After including these variables in the same model, maternal age, paternal age, endometrial thickness, and β-hCG were still independently associated with the risk of EPL, whereas the number of retrieved oocytes, duration of infertility, number of metaphase-2 oocytes, E2/P, and secondary infertility rate were no longer related (**Table 2**). AFC, with no significant correlation in the univariate analysis, was notably correlated in the multivariate analysis. The association between β-hCG levels and the risk of EPL explored in different logistic regression models is presented in **Table 3**. The total population was divided into four groups based on β-hCG levels. Compared to those with β-hCG <412.9 IU/L (quartile 1), women with quartile 2 (412.9–790.8 IU/L), quartile 3 (790.9–1390 IU/L), and quartile 4 (>1390 IU/L) had a significant decrease in risk of EPL. The adjusted odds ratios(ORs) remained similar to those in the unadjusted model after adjusting for maternal age, paternal age, number of retrieved oocytes, infertility duration, maternal BMI, total AFC, type of infertility, number of metaphase-2 oocytes, endometrial thickness, and E2/P.

**Table 2:** The risk factors associated with the incident early pregnancy loss in fresh IVF/ICSI cycles.

Variables	Univariable		Multivariable	
	OR (95%CI)	P value	OR (95%CI)	P value
Maternal age (y)	1.0781 (1.0612, 1.0954)	< .01	1.0423 (1.0158, 1.0696)	< .01
Paternal age (y)	1.0562 (1.0426, 1.0700)	< .01	1.0229 (1.0018, 1.0445)	< .05
Maternal BMI (kg/m2)	1.0274 (1.0053, 1.0499)	< .05	0.9910 (0.9666, 1.0161)	.48
Infertility duration (y)	1.0390 (1.0161, 1.0625)	<.01	1.0004 (0.9747, 1.0268)	.98
Infertility type				
Primary	1.0		1.0	
Secundary	1.2607 (1.0996, 1.4454)	< 0.01	1.0206 (0.8666, 1.2020)	0.81
AFC	0.9902 (0.9800, 1.0004)	.06	1.0187 (1.0055, 1.0321)	< .01
Number of retrieved oocytes	0.9749 (0.9641, 0.9859)	< .01	0.9642 (0.9277, 1.0021)	.06
Number of metaphase-2 oocytes	0.9774 (0.9662, 0.9888)	< .01	1.0231 (0.9840, 1.0638)	.25
Endometrial thickness (cm)	0.5633 (0.4055, 0.7826)	< .01	0.5669 (0.3950, 0.8136)	< .01
E2/P	0.9466 (0.9107, 0.9839)	<.01	0.9683 (0.9292, 1.0090)	.13
β-hCG (IU/L)	0.9987 (0.9985, 0.9988)	< .01	0.9987 (0.9986, 0.9989)	< .01

AFC, total antral follicle count; BMI, body mass index; OR, odds ratio; CI, confidence interval.

**Table 3:** The association between *β*-hCG level and the risk of early pregnancy loss occurrence.

hCG quartiles (IU/L)	Model 1	Model 2	Model 3
	Adjusted OR (95%CI)	Adjusted OR (95%CI)	Adjusted OR (95%CI)
<412.9	1.0	1.0	1.0
412.9-790.8	0.26 (0.22, 0.31)*	0.26 (0.22, 0.31)*	0.27 (0.22, 0.33)*
790.9-1390	0.18 (0.15, 0.22)*	0.19 (0.15, 0.23)*	0.19 (0.15, 0.24)*
>1390	0.11 (0.09, 0.14)*	0.11 (0.09, 0.14)*	0.11(0.09, 0.14)*
P for trend	< 0.001	< 0.001	< 0.001

CI, confidence interval; OR, odds ratio.

Model 1:unadjusted.

Model 2:adjusted for maternal age, paternal age, number of retrieved oocytes, duration of infertility, and maternal BMI.

Model 3:adjusted for maternal age, paternal age, number of retrieved oocytes, duration of infertility, maternal BMI, total AFC, type of infertility, number of metaphase-2 oocytes, endometrial thickness, E2/P.

*P < 0.001.

**Figure 3:**
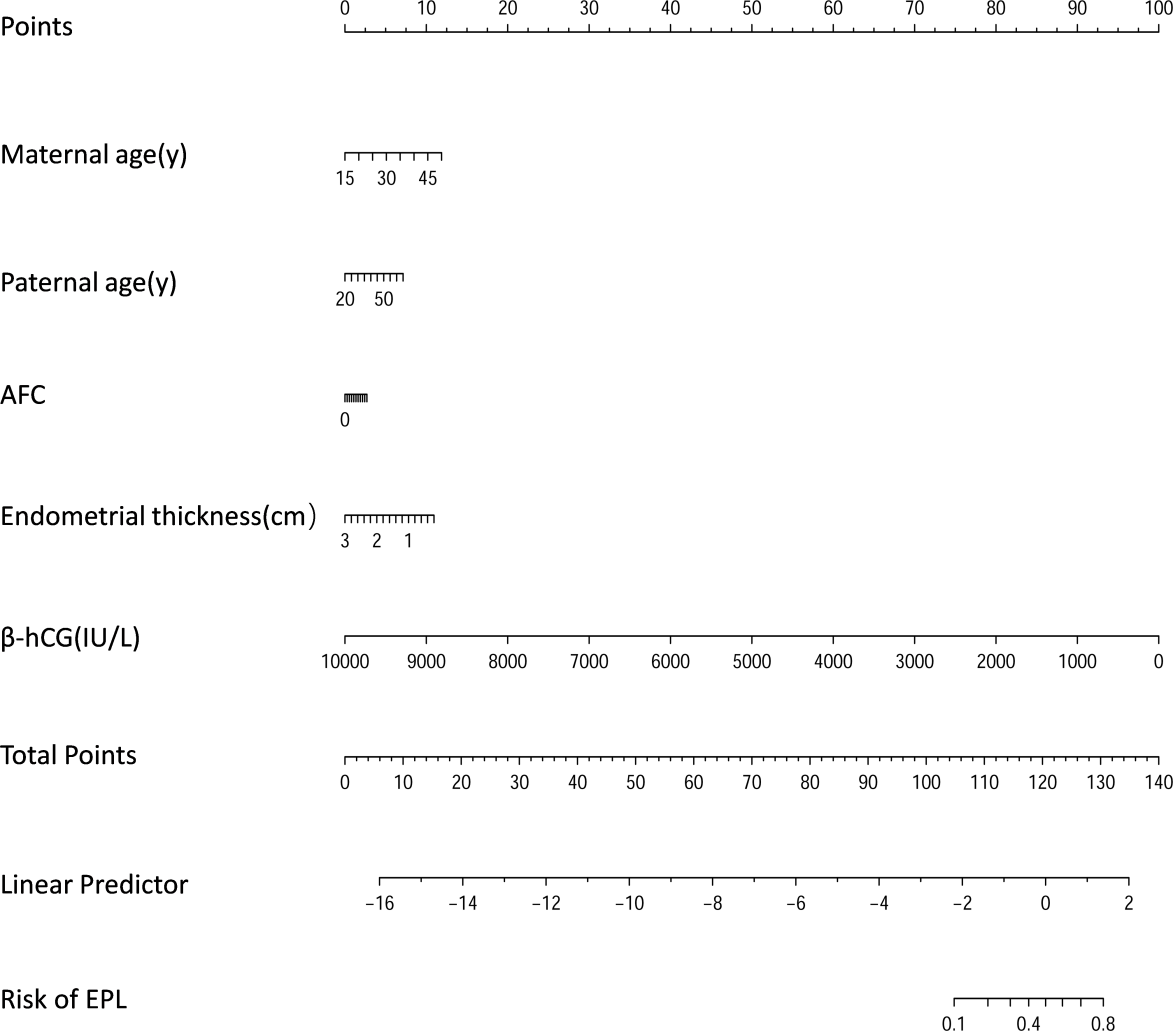
Nomogram for predicting the risk of EPL. AFC, total antral follicle count.

The prediction model was created as a nomogram using maternal age, paternal age, total AFC, endometrial thickness, and β-hCG (**Figure 3**). Considering the span of the factors, β-hCG was dominated as the strongest factor in the prediction model.

**Figure 4:**
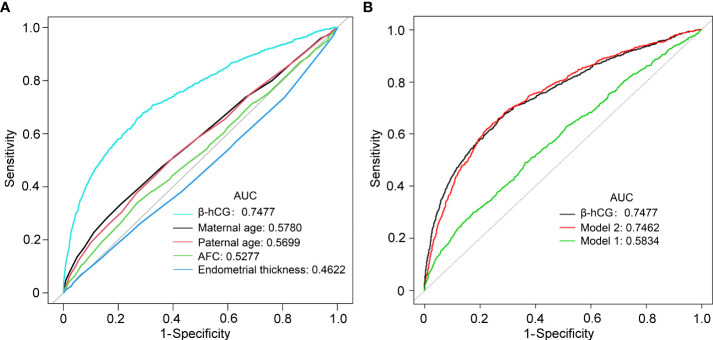
Receiver operating characteristic (ROC) curves comparing the potential of different variables to predict early pregnancy loss. **(A)** The prediction of individual variables. **(B)** Predictive ability of different models of early pregnancy loss. Model 1 included maternal age, paternal age, AFC, and endometrial thickness; Model 2, model 1 plus β-hCG.

We performed ROC curve analyses to explore the predictive value of serum β-hCG levels and other risk factors for EPL (**Figure 4**). The AUC for the β-hCG level was 0.7477, which was significantly higher than maternal age, paternal age, total AFC, and endometrial thickness (**Figure 4A** and **Table 4**). The best β-hCG threshold value for predicting EPL based on Youden’s index algorithm in the ROC curve was 542.45 IU/L, with a specificity of 71.13%, a sensitivity of 67.71%, positive predictive value (PPV) of 28.80%, and negative predictive value (NPV) of 92.74%. Furthermore, two prediction models incorporating other risk factors were established. Model 1 incorporated other risk factors except for β-hCG, including maternal age, paternal age, AFC, and endometrial thickness; the model’s AUC was 0.5834 (95%CI:0.563–0.604). After including β-hCG into the model, the AUC of the new model (model 2) increased from 0.5834 to 0.7462(95% CI 0.708-0.746; P < 0.001). Model 2’s PPV and NPV were 31.91% and 92.29%, respectively (**Figures 4B**, **Table 4**). Therefore, we may naturally conclude that serum β-hCG levels can be a strong independent predictor of EPL.

**Table 4:** Accuracy of different variables and models in IVF/ICSI clinic pregnancy to predict early pregnancy loss.

	AUC	95%CI	P value	Best threshold	Specificity (%)	Sensitivity (%)	PPV (%)	NPV (%)
β-hCG (IU/L)	0.7477	0.7296-0.7657		542.45	71.13	67.71	28.80	92.74
Maternal age (y)	0.5780	0.5576-0.5983	< .001	33.50	79.00	33.47	21.75	87.19
Paternal age (y)	0.5699	0.5498-0.5901	< .001	32.50	59.56	51.44	18.13	87.57
AFC	0.5277	0.5072-0.5482	< .001	10.75	73.24	33.75	17.99	86.41
Endometrial thickness (cm)	0.4622	0.4421-0.4824	< .001	1.48	93.49	6.97	15.78	85.17
Model 1	0.5834	0.5626-0.6043	< .001	-1.73	63.62	48.95	18.78	87.88
Model 2	0.7462	0.7279-0.7645	.815	-1.39	77.54	61.9	31.91	92.29

AUC, area under the curve; CI, confidence interval; NPV, negative predictive value; PPV, positive predictive value.

Model 1 included maternal age, paternal age, No. of retrieved oocytes, AFC and endometrial thickness;.

Model 2, model 1 plus β-hCG.

P values express the significance of the differences between β-hCG and other variables or the two models.

## Discussion

In this study, we demonstrated that the serum β-hCG levels were significantly associated with the risk of EPL in fresh IVF/ICSI cycles, independent of other risk factors, which included the maternal age, paternal age, number of retrieved oocytes, the duration of infertility, maternal BMI, total AFC, number of metaphase-2 oocytes, endometrial thickness, E2/P, type of infertility, and fertilization methods. In addition, β-hCG exhibited a significant AUC than the other risk factors for predicting EPL. More importantly, our findings suggest that β-hCG had a more significant predictive value than the model including other risk factors, and incorporating other risk factors into the β-hCG model could not enhance the ability to predict EPL.

In the early stage of pregnancy, hCG is one of the first signals provided by the embryo to the mother. On one hand, hCG rescues the corpus luteum and guarantees the ongoing production of progesterone (11). On the other hand, hCG may signal to the endometrium of future embryo implantation, promote trophoblast cell differentiation and growth, and establish placental villous structures. In addition, hCG promotes immunologic tolerance and angiogenesis at the maternal-fetal interface (11). Apart from these fundamental roles in early pregnancy, hCG is critical in accelerating the invasion of trophoblastic cells into the endometrium by over-modulating ERK and AKT signals (18). One study showed that cytokines related to hCG after 4 weeks of pregnancy were significantly altered in women with spontaneous miscarriage (19). Thus, lack of hCG secretion may be relevant to EPL (20) Meanwhile, many researchers have explored the clinical effect of hCG intrauterine infusion in assisted reproduction patients, with inconsistent results (21–24). However, the pathophysiological mechanism linking hCG to EPL has not yet been established.

Several studies have established an association between serum hCG levels after embryo transfer and EPL. A low serum hCG level is generally considered to indicate a high risk of EPL. Early in pregnancy, serum hCG levels reflect the function of villous trophoblasts, allowing pregnancy outcomes to be predicted. Hu et al. observed that the hCG level 14 days after the transfer was significantly higher in the live birth group than in the miscarriage group, especially EPL, in a large retrospective cohort study, however, the predictive cut-off value was not determined (25). Lawler et al. (26) reported that the mean β-hCG level 12 days after embryo transfer was 263 ± 207 mIU/mL in the group with positive fetal cardiac motion. Zhang et al. (27)reported that the serum β-hCG level in live births was significantly higher than in spontaneous miscarriage (596.8 IU/L vs 357.15 IU/L; P < 0.001). This result is consistent with our findings, but a definite cut-off value for EPL prediction remains unestablished. However, none of these studies explored whether serum β-hCG levels combined with other related factors increased EPL prediction.

Many researchers have studied the risk factors for EPL in ART treatment throughout the years, including maternal age, paternal age, maternal BMI, and endometrial thickness. Most clinical studies have shown that the pregnancy loss rate increases with maternal age, particularly beyond the age of 35 (28, 29). In elderly women, the underlying cause seems to be decreased follicle reserves and chromosomal aneuploidy (30). Studies examining the association between paternal age and EPL are controversial (31, 32). Wang et al. discovered that in couples undergoing ART therapy, a relatively young paternal age was associated with an increased risk of chromosomal aberration-related miscarriages (33). Most previous studies (31, 32) concluded that paternal age did not affect the EPL rate after IVF/ICSI. A recent meta-analysis suggested that increased paternal age raises the risk of spontaneous miscarriage (34). In the present study, paternal or maternal age was significantly associated with EPL in the univariate and multivariate analyses, but the predictive value was poor (AUC < 0.6) (**Table 2**).

A series of studies have concluded that endometrial thickness has a strong correlation with pregnancy outcomes. Gallos et al. reported that the endometrial thickness was strongly correlated to pregnancy loss and that an optimal endometrial thickness threshold of ≥10 mm minimized pregnancy loss (35). In another study, K. E. et al. considered that when the endometrial thickness was <8 mm, the pregnancy loss rates in fresh cycles rose with each millimeter decline (36). Conversely, other researchers have considered that the endometrial thickness has little bearing on pregnancy outcomes. Shakerian et al. (37) found that the endometrial thickness was not predictive of live births in either fresh or frozen-thawed embryo transfer cycles. In our study, the endometrial thickness showed a significant relationship with EPL in both univariate and multivariate analyses, although the AUC was only 0.4622, implying a poor predictive value. In addition, a few published studies have demonstrated an association between AFC and pregnancy outcomes, particularly the EPL rates. A recent meta-analysis indicated an association between low AFC and miscarriage incidence; however, only 3 small-sample retrospective studies were included (38).

The strengths of the present study lie in the larger cohort assessed, serum β-hCG measurements, and various clinical risk factors. The sample size was sufficiently large to answer the study questions. These measurements were performed in the same laboratory using the same equipment. It significantly reduced the variation caused by laboratory testing. In this study, the exclusion criteria were strictly met. Our study, however, has three limitations. First, this was a retrospective study. Second, although multiple pregnancies were excluded from this study, multiple embryo transfers were included, which may have interfered with the outcome. Third, the current serum β-hCG prediction value for EPL in this study performed moderately, with a specificity of 71.13% and a sensitivity of 67.71%. Although serum β-hCG was important in excluding the risk of EPL in fresh IVF/ICSI cycles due to its high NPV(92.74%), the false-positive rate associated with low PPV(28.8%) should be noted. Hence, it is necessary to improve the ability to predict EPL beyond the current capabilities.

In conclusion, the present study suggests that serum β-hCG levels 14 days after transfer are an independent and significant predictor of EPL in fresh IVF/ICSI cycles and that combining other risk factors and serum β-hCG levels did not improve the prediction effect.

## Data availability statement

The raw data supporting the conclusions of this article will be made available by the authors, without undue reservation.

## Ethics statement

This study was approved by the Ethics Committee of the First Hospital of Lanzhou University [Ethic no. LDYYSZLLKH2021-12]. The need for written informed consent was waived off by the institutional review board.

## Author contributions

LW designed the study, analyzed the data, and drafted the manuscript. YJ, PJ, RZ, and HS participated in data acquisition and analysis. XM and MG participated in critical discussion and revision of the article. XZ and LZ supervised the study, including the design, procedures, and revisions of the article. All authors contributed to the article and approved the submitted version.

## Funding

This work was supported by the National Natural Science Foundation of China (81960279), Gansu Youth Science and Technology Project (2019-0406-JCC-0138), Gansu Innovation Base and Talent Program (21JR7RA391), and the 1st Hospital of Lanzhou University Fund (ldyyn2020-4).

## Conflict of interest

The authors declare that the research was conducted in the absence of any commercial or financial relationships that could be construed as a potential conflict of interest.

## Publisher’s note

All claims expressed in this article are solely those of the authors and do not necessarily represent those of their affiliated organizations, or those of the publisher, the editors and the reviewers. Any product that may be evaluated in this article, or claim that may be made by its manufacturer, is not guaranteed or endorsed by the publisher.
